# Metabolomic Characterization of *Phoradendron brachystachyum* Mistletoe and In-Silico and In-Vitro Investigation of Its Therapeutic Potential in Metabolic Disorders

**DOI:** 10.3390/plants12142729

**Published:** 2023-07-22

**Authors:** Luis Aurelio Montoya-Inzunza, Aldo Moreno-Ulloa, Rommel A. Carballo-Castañeda, Jorge Xool-Tamayo, Laura Aracely Contreras-Angulo, Nayely Leyva-López, Marilena Antunes-Ricardo, Jose Reyes Gonzalez-Galaviz, José Basilio Heredia, Erick Paul Gutiérrez-Grijalva

**Affiliations:** 1 Centro de Investigación en Alimentación y Desarrollo, A.C., Carretera a Eldorado Km 5.5, Col. Campo El Diez, Culiacán 80110, Mexico; lmontoya221@estudiantes.ciad.mx (L.A.M.-I.); lcontreras@ciad.mx (L.A.C.-A.); nayelyleyvalop@gmail.com (N.L.-L.); jbheredia@ciad.mx (J.B.H.); 2 MS2 Laboratory, Biomedical Innovation Department, Centro de Investigación Científica y de Educación Superior de Ensenada, Ensenada 22860, Mexico; amoreno@cicese.mx (A.M.-U.); rcarballo@cicese.edu.mx (R.A.C.-C.); jxool@cicese.mx (J.X.-T.); 3 Posdoc CONAHCYT-Centro de Investigación en Alimentación y Desarrollo, A.C., Carretera a Eldorado Km 5.5, Col. Campo El Diez, Culiacán 80110, Mexico; 4 The Institute for Obesity Research, Tecnológico de Monterrey, Av. Eugenio Garza Sada 2501 Sur, Monterrey 64849, Mexico; marilena.antunes@tec.mx; 5 School of Engineering and Science, Tecnologico de Monterrey, Av. Eugenio Garza Sada 2501 Sur, Monterrey 64849, Mexico; 6 Cátedras CONAHCYT—Instituto Tecnológico de Sonora, Ciudad Obregón 85000, Mexico; jrgonzalez@conacyt.mx; 7 Cátedras CONAHCYT—Centro de Investigación en Alimentación y Desarrollo, A.C., Carretera a Eldorado Km 5.5, Col. Campo El Diez, Culiacán 80110, Mexico

**Keywords:** metabolomics, chemoinformatics, mistletoe, *Phoradendron*, pancreatic lipase, antioxidant

## Abstract

Plants of the *Phoradendron* genus have been traditionally used for their lipid- and glucose-lowering effects. However, the compounds responsible for these effects and the overall chemical profile of these plants have not been thoroughly investigated. We aimed to characterize the metabolome of leaves, stems, and aerial parts of the *Phoradendron brachystachyum* plant. We used mass spectrometry and colorimetric screening techniques (with various solvents) to identify and characterize the metabolites present. We also evaluated the antioxidant (FRAP, ORAC, TEAC, and DPPH assays) and inhibitory effects on pancreatic lipase and α-glucosidase enzymes of hydrophilic extracts. Furthermore, we compared the molecular fingerprints between the identified metabolites and FDA-approved drugs to gain insights into the metabolites that might be responsible for the observed effects on enzymes. Our findings revealed the presence of 59 putative metabolites, primarily flavonoids. However, we also hint at the presence of peptide and carbohydrate derivatives. The leaf extracts demonstrated the most promising metrics across all assays, exhibiting strong antioxidant and enzyme inhibitory effects as well as high levels of phenolic compounds, flavonoids, and tannins. Fingerprint analysis suggested potential peptide and carbohydrate metabolites as pancreatic lipase and α-glucosidase inhibitors. Overall, our study provides evidence on specific metabolites in *Phoradendron brachystachyum* that could be responsible for the therapeutic effects noted in obese and type 2 diabetes subjects.

## 1. Introduction

Metabolic syndrome, a condition associated with an increased risk of cardiovascular disease, is characterized by the presence of four factors: high blood pressure, obesity, hyperglycemia, and dyslipidemia [1,2]. The worldwide prevalence of metabolic syndrome is rising, leading to significant healthcare expenses and increased cardiovascular mortality [3,4]. In addition, overweight and obesity are closely linked to hypercholesterolemia and diabetes, which can result in complications and damage to various tissues [5].

Oxidative stress, caused by an imbalance between the levels of antioxidant compounds and reactive oxygen species (ROS), contributes to lipid, protein, and DNA damage. Elevated ROS levels also trigger inflammation, leading to comorbidities like diabetes, cancer, and cardiovascular diseases [6,7,8,9].

In Mexico, many plant species are used in traditional medicine to treat diseases. However, comprehensive information about their chemical composition is limited for most species. Therefore, such studies are essential to ensure safety, quality control, and understanding of their bioactive properties [10,11].

One prominent plant used in Mexico is the American mistletoe from the *Phoradendron* genus. Recent studies have focused on characterizing the metabolites and evaluating the properties of various species of *Phoradendron* mistletoe, including *P. piperoides* (with observed antioxidant and analgesic properties) [12], *P. reichenbachianum* (linked to hypoglycemic and hypolipemic properties due to its terpene composition) [13], *P. velutinum* (demonstrating hypolipemic properties by reducing adipogenesis markers) [14], and *P. californicum* (possess antioxidant properties attributed to phytochemicals like phenols, coumarins, flavonoids, and tannins) [15,16,17]. *Phoradendron brachystachyum*, commonly known as “toji”, is a hemiparasitic plant used in México to treat hypercholesterolemia, hyperglycemia, and the common cold. The bioactive effects of mistletoe have been linked to terpenes such as moronic acid [14]. However, the characterization of other mistletoe compounds, including phenolic acids and flavonoids, remains incomplete, thereby limiting our understanding of the chemical space of this type of plant. Understanding the chemical composition and the mechanism through which these metabolites exert their bioactivity is crucial, as various mistletoe species have shown potential in controlling diabetes- and obesity-related disorders by acting as antioxidants (oxidative stress reducers) and selectively inhibiting key metabolic enzymes (e.g., pancreatic lipases and α-glucosidase).

Therefore, this study aimed to analyze the metabolomic composition of the American mistletoe, *Phoradendron brachystachyum*, and evaluate its potential in inhibiting enzymes associated with metabolic syndromes, such as pancreatic lipase and α-glucosidase.

## 2. Results and Discussion

### 2.1. Phytochemical Screening

The results of phytochemical screening assays showed the presence of flavonoids, tannins, and coumarins as major compounds in leaf and stem extracts of *P. brachystachyum* in hexane, methanol, and water extracts (Table 1). These results are similar to what is reported by Vasconcellos et al. [12] and Iloki-Assanga et al. [18] in hydrophilic extracts of *P. piperoides* and *P. californicum*, respectively. These studies mainly highlight tannins, flavonoids, and coumarins as the most abundant groups of phytochemicals.

Since phytochemicals may be differentially distributed in plant tissues, we analyzed the phytochemicals present in the stem and leaf methanolic extracts of mistletoe. In this sense, our results show no apparent difference in the abundance of the compounds detected by phytochemical screening. However, it is also observed that a higher abundance of tannins, flavonoids, and coumarins was obtained in methanol and water extracts of medium and high polarity, respectively; this may be due to the polar nature of the compounds [19,20].

The high content of tannins, flavonoids, and coumarins found in the leaf and stem could be due to the biological functions of these molecules, such as defense and pollinator attractors, as they are exposed to external factors such as predators, fungi, and bacteria, as well as UV light [21,22,23]. Furthermore, being a parasitic plant, the mistletoe stem is attached to the stem of the host plant, forming connections by which it extracts nutrients to survive; in consequence, as a defense mechanism, there is a phenomenon known as allelopathy, in which the host plant synthesizes compounds in response to this, and in turn, the parasitic plant constantly generates compounds to counteract such a defense response, so phytochemical compounds will likely concentrate on the stem [24]. Some of the most common phenolic compounds in this mistletoe genus are *p*-hydroxybenzoic, protocatechuic, gentisic, and gallic acids. Some common coumarins include warfarin and aesculetin. While the most common flavonoids are apigenin, luteolin, and glycosyl-flavones C-6 and C-8 [22].

### 2.2. Metabolomic Analysis

**Figure 1:**
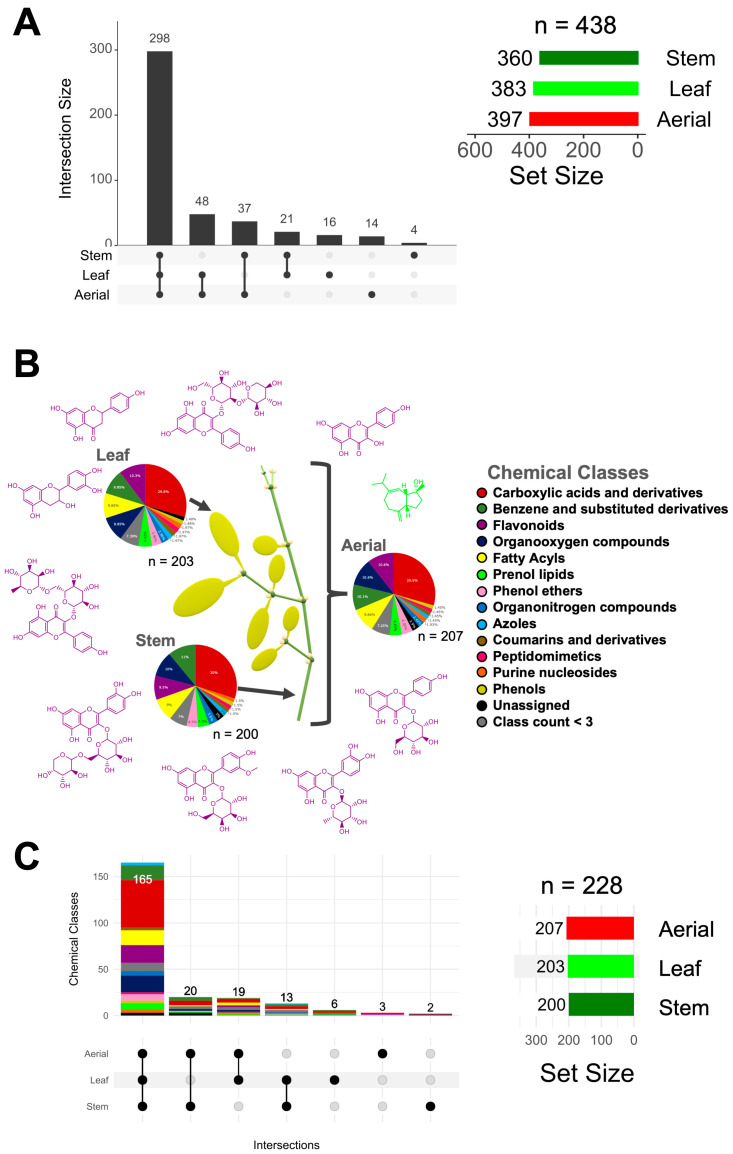
Metabolomics profiling of the mistletoe plant by mass spectrometry-based untargeted metabolomics. (**A**) The overlapping pattern of the 438 aligned features (at the MS1 level) was visualized using UpSet plotting (UpSetR version 1.4.0), wherein the bar height (intersection size) denotes the proportion of the features assigned to each unique (singular black dot or circle) or shared plant’s anatomical site (multiple linked black dots or circles). The Set Size (colored bars) denotes the total number of features detected by the site. (**B**) Metabolite chemical class distribution by site. Representative putatively identified metabolites (purple = Flavonoids, green = Prenol lipids) are shown. (**C**) Chemical characterization of the overlapping features among sites.

A total of 438 aligned features (at the MS1 level) among *P. brachystachyum* anatomical parts (leaf, stem, and aerial) were generated by MZmine software (version 2.53) and remained after several filtering steps. To provide a first comparative metabolomic analysis among the plant’s anatomical parts, we determine the overlapping pattern of each metabolome (at the feature level) with each other by UpSet plotting. The part with the highest number of unique features detected was the leaf (16 features), followed by the aerial parts (14) and the stem (4) (Figure 1A). The sites that shared more features were the leaf-aerial parts, followed by the stem-aerial parts and the stem-leaf.

A global chemical class profiling analysis revealed a wide range of classes on the plant’s anatomical sites (228 annotations through CANOPUS (MSI classification, level 3)), with flavonoids among the three most abundant chemical classes after carboxylic acids and derivatives and benzene and substituted derivatives (Figure 1B). The same chemical classes were identified in the leaf and stem, hinting at a chemically similar global metabolome. To further profile the metabolomes at the structure level, we employed automatic spectral matching against GNPS’s public spectral libraries (MSI classification, level 2) and in-silico predictions (MSI classification, level 3), whereby we could annotate 59 compounds with high confidence. Within the chemical classes, flavonoids were the principal class with identified molecular structures (Figure 1B and Table 2), and while this type of metabolite was present in all plant parts, other chemically diverse metabolites were also shared among leaf, stem, and aerial, for which no putative molecular structures were retrieved (Figure 1C). Only a few metabolites were either uniquely present in the leaf or stem; the majority were detected in both sites.

Flavonoids are a group of secondary metabolites with many associated biological effects, including antioxidant, anti-inflammatory, anti-viral, and anti-cancer properties [25]. Nonetheless, not all metabolites can pass the gastrointestinal tract barrier and therefore exert a systemic effect [26]. Thus, to gain insight into the potential impact on human health of the metabolites identified in *Phoradendron brachystachyum*, we predicted their bioavailability using the SwissADME online platform [27,28,29]. However, due to the high hydrophilic properties of variously identified flavonoid glycosides, their predicted bioavailability was low, while for non-glycoside (i.e., aglycone) flavonoids, the opposite was true (Table 2). However, flavonoids can target biomolecules along the GI tract and interact with the microbiota, thereby modulating human health despite poor bioavailability [30,31].

### 2.3. Molecular Fingerprints-Based Visual Comparison among Phoradendron brachystachyum Metabolites and FDA-Approved Drugs

**Figure 2:**
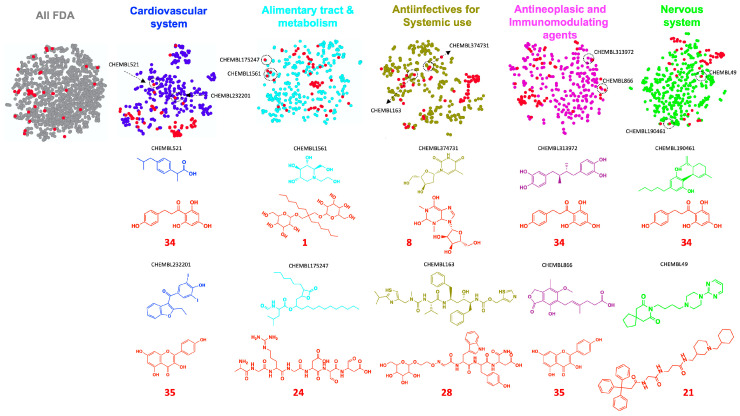
Visual representation of the chemical structure similarity among FDA-approved drugs and the identified metabolites in *Phoradendron brachystachyum*. Figures were generated with t-SNE using the FragFp descriptor. Y (t-SNE 2) and x (t-SNE 1) axes are omitted to enhance data visualization. Each node represents a molecular structure, and its color (red for the metabolites identified in *Phoradendron brachystachyum*) indicates its origin (FDA-approved or plant). The red numbers and molecular structures correspond to the IDs and identified metabolites described in Supplementary Table S1, respectively. ChEMBL codes correspond to the IDs of the FDA-approved drugs retrieved from https://www.ebi.ac.uk/chembl/g/#search_results/all/query=FDA, accessed on 23 June 2023). Black intermittent circles indicate examples of similar molecules among the FDA-approved plant metabolites. The color of the molecular structure is associated with the subtype or code description of FDA-approved drugs. Structures were drawn using ChemDraw Professional software (version 16.0.1.4).

To provide information on the potential bioactivities of the identified metabolites in *Phoradendron brachystachyum*, we compared their molecular structure similarity (based on molecular fingerprints) to those of the FDA-approved drugs contained within the ChEMBL database using a t-SNE visualization. To facilitate our analysis, we stratified the visualizations according to the ATC codes of the drugs. We noted that various metabolites shared the chemical space with the drugs. In Figure 2, we show all the visual comparisons and denote representative FDA drugs and metabolites that shared the chemical space due to their molecular structure similarities. Particularly, we noted that kaempferol and phloretin clustered with various drugs on several comparisons. On the other hand, metabolites 1 and 24 clustered with miglitol (α-glucosidase inhibitor) and orlistat (a pancreatic lipase inhibitor), respectively. These findings agree with the enzyme inhibition assays and suggest that, at least, metabolites 1 and 24 are mediating such inhibitory effects. Overall, our results suggest that *Phoradendron brachystachyum*-based extracts could mediate biological effects on multiple systems and organs.

In addition, we analyzed these metabolites in the tool SwissTargetPrediction (http://www.swisstargetprediction.ch/, accessed on 19 May 2023) to determine what possible enzyme interactions our metabolites have. We also analyzed their physicochemical properties to predict their drug-likeness by passive absorption; 16 metabolites can be bioavailable (Table 2).

### 2.4. Total Phenolic and Flavonoid Content

Mistletoe leaves exhibited the highest concentration of phenolic compounds, followed by the aerial parts and the stem (*p* < 0.05) (Table 3). These results are comparable to those shown by Fitrilia, Bintang, and Safithri [32], where 85.6 mg GAE/g sample equivalents were reported in hydrophilic extracts of *Dendrophthoe pentandra* L. Similarly, in the results reported by Kang et al. [33], a total phenolic content of 65.78 mg GAE/g sample was observed in hydrophilic extracts of *Korthalsella japonica* Engl. Additionally, a content of 203.78 ± 1.95 mg GAE/g was also reported in hydrophilic extracts of *Phragmanthera capitata* leaves, according to Ohikhena, Wintola, and Afolayan [34].

The most flavonoid-rich part was the leaf, compared to the stem and the aerial parts (*p* < 0.05) (Table 3). These results are comparable to those obtained by Kang, Park, Kim, Yang, Kim, and Kim [33], where 16.23 mg rutin equivalents/g sample were reported in hydrophilic extracts of *Korthalsella japonica* Engl. Ohikhena, Wintola, and Afolayan [34] reported values of 407.90 mg QE/g sample of hydrophilic extracts in *Phragmanthera capitata* leaves.

### 2.5. Content of Total, Condensed, and Hydrolyzable Tannins

The highest content of total (TT), hydrolyzable (HT), and condensed (CT) tannins is shown in the leaf and aerial part extracts (Table 4). The stem extract showed the lowest concentration of tannins. This is comparable to the results obtained by Ohikhena, Wintola, and Afolayan [34], where they assessed the number of condensed tannins present in hydrophilic extracts of *Phragmanthera capitata* and obtained 194.00 mg CE/g sample.

Polyphenols such as phenols, flavonoids, and tannins are secondary metabolites that plants synthesize as protection against biotic and abiotic stresses. These compounds usually accumulate at higher concentrations in plant leaves and fruit peel, as these are the parts of the plant that are most exposed to this type of stress [35]. The amount of polyphenols extracted from a plant matrix depends on the type of solvent used; phenols, flavonoids, and tannins are easy-to-extract metabolites in hydrophilic solvents thanks to their polar nature [34,36]. These polyphenols protect the plant against oxidative stress and various environmental factors, so it is common to find them in them; flavonoids, for example, function as pigments and antioxidants in plant metabolism. On the other hand, tannins have antioxidant potential and also function as toxins or anti-nutrimental compounds when binding with proteins, thus preventing them from doing their function; in this way, they also function as repellents for predators that prevent the consumption of plants that own them [37].

### 2.6. Antioxidant Capacity

The DPPH (2,2-diphenyl-1-picrylhydrazyl) assay evaluates the antioxidant capacity of natural compounds and plant extracts. The change from intense purple to dim yellow indicates the antioxidant potential of samples evaluated for their ability to donate electrons. This trial is sensitive to detecting active compounds at low concentrations [31]. The results of the antioxidant capacity by the DPPH method show that the aerial parts had the highest antioxidant capacity over the stem (*p* < 0.05) (Table 5). However, the antioxidant capacity of the leaf extract was not statistically different (*p* > 0.05) from that of the stem and the aerial parts. The IC_50_ of a standard ascorbic acid curve was calculated, as it is usually used as a comparison point. The results showed a much higher antioxidant capacity than this standard; this can be because of the high polyphenolic concentrations present in our mistletoe extract. Our results show that the entire plant has the highest in vitro reducing capacity, which has been reported to occur mainly by donating electrons to the radical DPPH relative to the stem, as it is observed that the use of the two parts together slightly increases its reducing capacity, thus presenting a synergistic effect between the leaf and stem phytochemicals of mistletoe. These results are comparable to studies conducted by Wang et al. [31], where they evaluated a methanolic extract of *Phoradendron* sp. and its antioxidant potential by the DPPH assay, obtaining an IC_50_ of 58.36 μg/mL in the aerial parts. Also, Fitrilia et al. [32] reported an IC_50_ of 11.4 μg/mL in *Dendrophthoe pentandra* L. leaf hydrophilic extracts. Ohikhena et al. [34] mentioned that hydrophilic leaf extracts of *Phragmanthera capitata* showed an IC_50_ of 67.2 μg/mL. The differences between the IC_50_ values between these mistletoes may be due to several factors, including the species difference of the plant, the host that is parasitizing the mistletoe, the method of processing, and the environmental stress to which they may have been subjected [35,36,37,38].

The TEAC (Trolox equivalent antioxidant capacity) assay is a spectrophotometric method like the DPPH method, based on measuring antioxidant capacity by reducing radical ABTS by electron donation, which is reflected through the radical color change from an intense blue/green to a less intense blue color [39].

The part with the highest antioxidant capacity in vitro was the leaf, followed by the stem and the aerial parts (*p* < 0.05) (Table 3). The IC_50_ of a standard ascorbic acid curve was calculated as a comparison point. These results are comparable to the study conducted by Wang et al. [32], who evaluated methanolic extracts of *Phoradendron* sp. and their antioxidant potential by the TEAC assay, obtaining an IC_50_ of 9.91 μg/mL in the complete plant. Similarly, in the study by Ohikhena, Wintola, and Afolayan [34], an IC_50_ of 6.8 μg/mL was obtained in hydrophilic extracts of *Phragmanthera capitata* leaves. The mistletoe extract showed a reducing power greater than or like those previously reported, and this may be due to the same factors previously mentioned in the DPPH test.

Unlike the trial with the radical DPPH, the compounds in the aerial parts did not have the same synergistic effect, so it could be theorized that the phytochemicals present in the mistletoe leaf have greater ease in donating electrons to the radical ABTS structure compared to those present in the stem, possibly related to an antagonistic effect between the phytochemical leaf and stem compounds in the inhibition of the radical ABTS. Some studies report that this antagonistic effect could be due to the formation of polymers among plant compounds, which may increase or decrease their antioxidant capacity [40]. However, more studies are needed on the formation of polymers in this species to support this phenomenon.

The ORAC assay shows us a way more attached to what happens in the organism on the antioxidant potential of molecules on free radicals through the donation of hydrogen ions (protons) since it simulates both the pH and the temperature that are naturally found in the human body and the generation of the peroxyl radical (only generated in humans in oxidative stress) [41]. The extracts from the leaf and aerial parts showed the highest antioxidant capacity in the ORAC assay. In contrast, the lowest antioxidant potential was found in the stem (Table 5), as we have already seen in previous results.

We can compare this with what is reported by VICAŞ et al. [42], who studied the variety *Viscum album* parasitizing *Malus domestica* with values similar to ours of 1870 and 1520 μmol TE/g sample in leaf and stem, respectively. They also carried out the same evaluation with the same variety but parasitizing a different host (*Hacer campestre*) and had results of 6330 and 5490 μmol TE/g sample for leaf and stem, respectively. We can see the antioxidant potential increase drastically when parasitizing another host, and this change can influence the composition and concentration of secondary metabolites in the same mistletoe [39].

The antioxidant capacity, measured by the FRAP assay, demonstrates the potential of the mistletoe extracts to reduce ferric ions to ferrous ions through electron donation [43]. The leaf extracts exhibited the highest antioxidant capacity, followed by the aerial parts and the stem extracts (*p* < 0.05) (Table 2). These results can be compared with those reported by Trifunschi et al. [44], who studied the variety Tristerix tetrandus with values of 135.32 μmol TE/g sample, which are lower than those of our mistletoe. The report by Majeed et al. [45] evaluated the best-known variety of mistletoe, *Viscum album,* with values of 500.63 μmol TE/g sample in the leaf, which were still lower than those obtained by our mistletoe.

Many of the cellular activities carried out in our body involve processes that generate reactive oxygen species (ROS); some of the most common are superoxide ions (O_2_^−^) and hydroxyl radicals (OH^−^). ROS in high amounts can cause oxidative stress, which can irreversibly damage various biomolecules, such as proteins, lipids, and DNA. Polyphenols possess in their structure aromatic rings that have one or more hydroxyl groups capable of donating electrons to this type of radical; therefore, they can prevent the excessive concentration of ROS in the body, avoiding damage to these biomolecules. In this sense, ROS are also correlated with the development of cardiovascular disease and carcinogenic processes [46].

Our results showed a lower IC_50_ of the mistletoe extracts against the radical ABTS with respect to the radical DPPH; this could be because the structure of the compounds present in our extracts has a greater affinity for electron donation towards the radical ABTS, unlike the affinity for DPPH [47].

In the same way, the ORAC and FRAP essays show us that our extracts have better reducing power by the donation of electrons than by the donation of protons, with better results in the FRAP essay than in the ORAC essay [41,42]. The leaf is the part that gets better results, which can be explained by the functions that every part of the plant has: the leaf is the part that has more functions like breathing, transpiration, photosynthesis, synthesis of different metabolites, and being more exposed to predators and external stress factors, while the stem has more structural functions and nutrient transport across the different parts of the plant, so the plant focuses more concentrations of this kind of metabolite in the leaf than the stem [37,48].

The antioxidant potential of mistletoe extracts can be attributed to tannins, the most abundant compounds in our samples; these molecules have a large number of hydroxyl groups attached to their structure, so their ability to reduce this type of radical is very high [49].

### 2.7. In Vitro Inhibition of Pancreatic Lipase and α-Glucosidase

As shown in Table 6, the inhibitory potential of hydrophilic extracts of *P. brachystachyum* expressed as % inhibition was assessed against carbohydrate metabolism-related enzymes, where we can see that at 15 μg/mL, the leaf had significantly more inhibition of α-glucosidase (32.73%) than the stem (29.36%) and aerial parts (24.13%), in the same way, the standard acarbose at 1 mM was analyzed as a positive control with inhibition of 58.33%. Likewise, the inhibitory potential of hydrophilic extracts of *P. brachystachyum* against pancreatic lipase was assessed, and we can see that at 50 μg/mL, the leaf had significantly more inhibition of the enzyme (71.44%) than the stem (49.11%) and aerial parts (51.71%), in the same way, that the standard orlistat at 60 μg/mL was analyzed as a positive control with inhibition of 72.38%. We can see that in both enzymes, the leaf was the part with more inhibitory potential.

**Figure 3:**
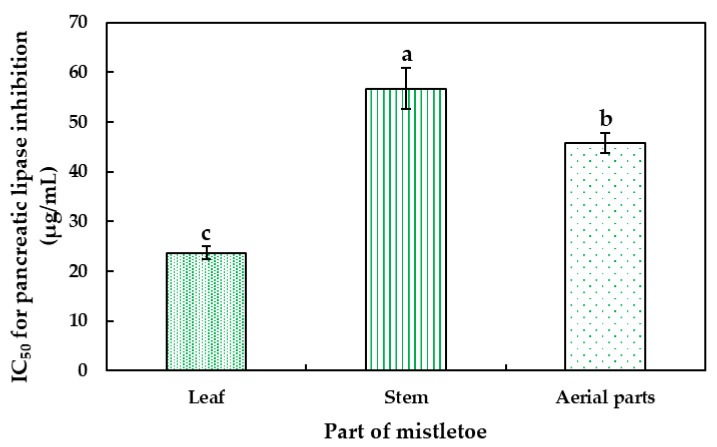
Effect of hydrophilic extracts from leaf, stem, and aerial parts of mistletoe (*P. brachystachyum*) on pancreatic lipase activity. Data are presented as mean ± standard deviation (n = 3). Different letters indicate significant differences between the parts by Tukey’s test (*p* < 0.05).

The results of the inhibitory potential of the hydrophilic extracts of *Phoradendron brachystachyum* on the pancreatic lipase enzyme showed (Figure 3) that the leaf exhibited higher inhibitory capacity (IC_50_ = 23.68 μg/mL), followed by the stem (IC_50_ = 56.71 μg/mL), and the aerial parts (45.74 μg/mL). These results can be compared with those obtained by Lee et al. [50], where they assessed the inhibitory potential on pancreatic lipase of the stem and leaf of *Sorbus commixta* and aerial parts of *Viscum album* with IC_50_ of 29.6 μg/mL and 33.3 μg/mL, respectively.

### 2.8. In Vitro Inhibition of α-Glucosidase

**Figure 4:**
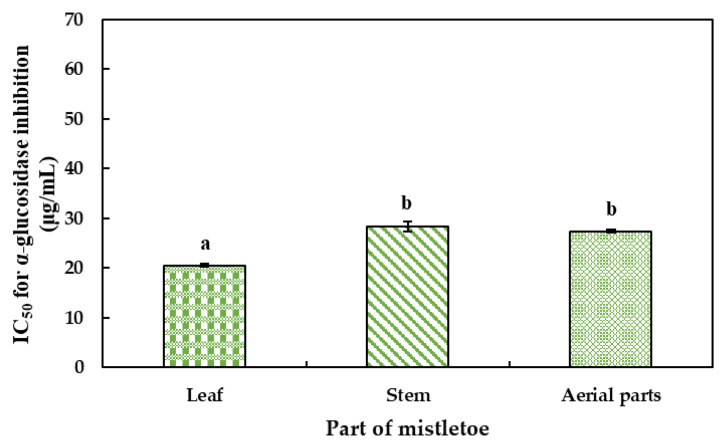
Effect of methanolic extracts from leaf, stem, and aerial parts of mistletoe (*P. brachystachyum*) on the α-glucosidase activity. Data are presented as mean ± standard deviation (n = 3). Different letters indicate significant differences between the parts by Tukey’s test (*p* < 0.05).

The results of the inhibitory potential of methanolic extracts of *Phoradendron brachystachyum* on the α-glucosidase enzyme (Figure 4) show that the leaf exhibited higher inhibitory capacity (IC_50_ = 20.51 μg/mL), followed by the stem (IC_50_ = 28.35 μg/mL), and aerial parts (27.40 μg/mL). These results can be compared with those obtained by Ohikhena et al. [51], where they assessed the inhibitory potential on α-glucosidase of the methanolic extracts of *Phragmanthera capitata* and reported an IC_50_ of 91.98 μg/mL [52,53,54,55].

Inhibition of enzymes involved in carbohydrate and fat metabolism is typically targeted in managing metabolic syndromes such as hyperglycemia, diabetes, and high cholesterol. The enzymatic inhibition observed in our assays can be attributed to the presence of phytochemical groups such as tannins, flavonoids, and coumarins. These compounds have one or more -OH radicals in their structure, which can act as both donors and hydrogen acceptors. In this way, these compounds can interact with different biomolecules, among which are proteins, forming hydrogen bridges [56].

During polyphenol-protein interactions through hydrophobic and hydrophilic bonds, some soluble and insoluble complexes can be formed. The formation of these complexes can affect the bioavailability and functions of both polyphenols and enzymes in the body, as well as the digestive capacity of enzymes. In many cases, the binding of polyphenols with the proline-rich protein chain of the enzyme causes the enzyme to unfold, thus modifying its structure and affecting its physiological activity.

Studies suggest that these protein interactions have a higher affinity for binding to galloyed and complex polyphenols compared to smaller molecules, which is attributed to the lower -OH distribution of smaller polyphenols, decreasing the number of interaction sites. Some of the complex polyphenols reported with these properties are tannins [46], which are present in large quantities and as a majority group in mistletoe hydrophilic extracts.

One of the most studied groups in pancreatic lipase and α-glucosidase inhibition is flavonoids like quercetin, which report increased inhibitory potential for pancreatic lipase when *C*-glycosyl flavones have two sugar residues in C6 [57]. It is theorized that the hydroxyl radical at position 3 (R2) and methoxyl at position 4 (R3) favor enzyme inhibition, being the group -OH with the most potential; also, flavan-3-ols have greater inhibitory potential when esterified compared to those who are not; and hydroxycinnamic acids have greater potential over hydroxybenzoic acids [35].

Other studies report competitive inhibition of pancreatic lipase by three polyphenols: gallic acid, epigallocatechin, and epigallocatechin gallate, some of which are present in the metabolome of *Phoradendron brachystachyum*. These have a common characteristic: the presence of traces of the galloyl group in their structure. Evidence in reports suggests the presence of traces of galloyl in flavan-3-ol structures as a prerequisite for lipase inhibition [58] and the presence of quercetin derivates like quercetin-3-O-β-d-arabinopyranosyl-(1→2)-β-D-galactopyranoside and quercetin-3-O-β-d-glucuronide.

It is reported that the chlorogenic and caffeic acids have little influence on the inhibitory activity of lipase, suggesting that glycosylation at the A ring of flavonoids could be responsible for the anti-lipase activity [59]. Based on reports, the greatest inhibitory potential of pancreatic lipase can be attributed to the presence of flavonoid-type polyphenols in mistletoe hydrophilic extracts. This coincides with the molecules identified in this study, belonging to the flavonol derivatives and having a high abundance of tannins [49,60]. Some mistletoes, like *Phragmanthera capitata* polyphenolic rich extracts, show a high inhibition of α-glucosidase and a low inhibition of α-amylase [53]. It is reported that hydroxylation in the B-ring of flavonoids significantly increases the inhibitory activity on α-glucosidase and hydroxylation in the C-ring significantly weakens the inhibitory potential. Some metabolites present in the *Phoradendron brachystachyum* methanolic extracts are reported as good α-glucosidase inhibitors, like catechin with a 2,3-cis structure, and a galloylated structure in C-1, ellagic acid quercetin [61], and hydrolysable and condensed tannins as well, with competitive inhibition by a hydrogen bond type mechanism [62,63,64].

## 3. Materials and Methods

### 3.1. Plant Material

The mistletoe (*P. brachystachyum*) was collected in Guamúchil, Sinaloa (25°44′42″ 108°05′22″) 07/2021, and was parasitizing mesquite trees (*Prosopis juliflora*). The plants were identified in the herbarium Jesús González Ortega of the Faculty of Agronomy of the UAS (FA-UAS-022352 and 013284). The aerial parts of the plant were separated into leaf, stem, and aerial parts. They were then washed in chlorinated water at 50 ppm and allowed to dry at 40 °C for 48 h in a forced-air convection drying oven. The dried sample was recovered, and leaf, stem, and aerial parts were ground individually in a Turf brand coffee mill to obtain the samples as a fine powder and then passed through a sieve of 600 μm. Finally, the fine powder was collected and stored.

### 3.2. Extraction of Phytochemicals

The methanol extraction was carried out in accordance with the methodology of Gutiérrez-Grijalva et al. [61], with some modifications. A sample of 0.2 g of mistletoe powder was mixed with 10 mL of methanol: water (8:2, *v*/*v*), then stirred for 2 h at 80 rpm. Subsequently, the samples were centrifuged (HERMLE Labortechnik GmbH type Z 36 HK and 221.22 rotor) at 10,000 rpm at 4 °C for 15 min. The supernatant of the extract was recovered, then the solvent was evaporated (pressure of 30-230 var and 40 °C, Multivapor P-6/P-12 BÜCHI Labortechnik AG), and the weight of the dry extract was obtained. The extract was resuspended in methanol (80%) or PBS to obtain a known concentration to subsequently recover 2 mL of each replica in opaque Eppendorf vials and store at 4 °C for further testing. The extracts were prepared in triplicate (n = 3). To determine the TEAC and DPPH assays, the extraction was performed as mentioned but using only pure methanol instead of aqueous methanol.

### 3.3. Phytochemical Screening

Phytochemical screening was performed by means of colorimetric reactions and precipitation according to the specific group of metabolites based on the methodology of Harborne [62]. The phytochemical groups were determined by the following methods: tannins by FeCl_3_ test, flavonoids by Shinoda, saponins by foam, coumarins by Baljet, alkaloids by Dragendorff, Mayer, and Wagner, terpenes by Liberman–Bouchard (triterpenes and steroid compounds), and Salkowiski (sterols and methyl sterols). Hexane, methanol, and water solvents were used to observe the groups present in solvents from lower polarity to greater polarity, respectively. A sample of 1 g (stem and leaf) was macerated with 20 mL of solvent for 24 h at 60 rpm, centrifuged at 10,000 rpm at 4 °C for 15 min, and the supernatant was recovered (n = 3).

#### 3.3.1. Tannin Determination

The tannins were determined by the FeCl_3_ method. One or two drops of FeCl_3_ (10%) were added to one mL of the extracts of *P. brachystachyum*. A blue or green coloration in the samples indicates the possible presence of hydrolyzable or condensate tannins, respectively.

#### 3.3.2. Flavonoid Evaluation

Fragments of Mg were added to 0.5 mL of *P. brachystachyum* extract. Then the mix was heated at 60 °C for 10 min. Afterward, concentrated HCl was added. A yellow or orange coloration indicates the presence of flavonoids.

#### 3.3.3. Saponin Evaluation

A sample of 0.5 mL of *P. brachystachyum* extract was mixed with 1 mL of boiling water and stirred vigorously. The test is positive if the foam is formed and held for at least 15 min. The abundance of saponins was determined by foam elevation.

#### 3.3.4. Coumarin Evaluation

Three mL of *P. brachystachyum* extract was added to a test tube, and then two or three drops of a 10% NaOH solution were added to observe yellow coloration or yellow precipitation in the presence of coumarins. The yellow coloration disappears when acidulated.

#### 3.3.5. Triterpene and Steroid Evaluation

The Liebermann–Burchard reagent was prepared by mixing 50 mL of pure ethanol, 5 mL of acetic anhydride, and 5 mL of sulfuric acid. The mixture was then cooled in an ice bath, and a drop of the Liberman–Burchard reagent was placed in the test tube with 1 mL of evaporated and resuspended extract in 1 mL of chloroform. The change of color to blue-green or red-violet confirms the presence of triterpenes or steroids. For Salkowski’s test, 1 mL of extract was evaporated, 1 mL of chloroform was added, a drop of Salkowski reagent was placed, and a purple-red-blue or red coloration was observed for the test to be positive.

#### 3.3.6. Alkaloid Evaluation

The alkaloids were determined by three tests: Wagner, Mayer, and Dragendorff. Wagner’s reagent was prepared by mixing an I_2_ solution in KI with 10 mL of distilled water. Mayer’s reagent was prepared from a solution of HgI_2_ at 2% and 0.5 g of KI in 10 mL of distilled water. Dragendorff was already prepared (SIG-44578-100ML-F, Merck KGaA, Darmstadt, Germany). For the determination, 1.5 mL of extract was evaporated, 0.25 mL of HCL was added to 10%, and the mixture was subsequently heated in a water bath for 10 min. Each of the tests was performed in triplicate. A reconnaissance reagent (Dragendorff, Mayer, and Wagner) was added to each tube. Mild or hasty turbidity (red or orange in Dragendorff, white or cream in Mayer, and brown in Wagner) demonstrates the possible presence of alkaloids.

### 3.4. Metabolomic Analysis

#### 3.4.1. Metabolite Extraction

We followed a protocol previously described by our group [63]. In brief, five mg of lyophilized sample (leaf, stem, and aerial parts) was extracted with 500 μL of a mixture of methanol:ethyl acetate:acetonitrile (ACN) (1:1:1) under sonication for 30 min at room temperature. The samples were then centrifuged at 14,000 rpm for 10 min at 40 °C, and 400 μL of the supernatant was transferred to an Eppendorf tube and dried down using a SpeedVac system. The dried extract was resuspended with a mixture of water and ACN (80:20, *v:v*) at a concentration of 1000 ng/μL. The samples were diluted at 300 ng/μL (water:ACN, 80:20, *v:v*), centrifuged at 14,000 rpm for 10 min at 4 °C, and the particle-free supernatant was recovered for liquid chromatography (LC) coupled to mass spectrometry (LC-MS/MS) analysis.

#### 3.4.2. Data Acquisition and Analysis by Liquid Chromatography (LC) Coupled to Mass Spectrometry (LC-MS/MS)

The samples (2 μL volume injection) were loaded into an LC Agilent 1260 Infinity device (Agilent Technologies, Inc., Santa Clara, CA, USA). The separation of the molecules was carried out using a ProtID-Chip-43 II column (C18, 43 mm, 300 Å, the particle size of 5 μm, equipped with an enrichment column of 40 nL). The mobile phases consisted of water with 0.1% formic acid (FA) as solution A and ACN with 0.1% FA as solution B. The gradient used consisted of a linear increase of 5% to 40% of B in 20 min and maintained with 40% of B for 5 min, another linear increase to 100% in 5 min and maintained with 100% of B for 5 min, and finally a change to 5% of A in 1 min and maintained with 5% of A for 9 min (column re-equilibration). The total run time was 40 min at a flow rate of 300 nL/min. Two blank samples (3 μL of 5% of mobile phase B) were acquired between extract sample injections to minimize potential carryover. The separated metabolites by LC were analyzed using an Agilent 6530A Q-TOF mass spectrometer (Agilent Technologies, Inc., Santa Clara, CA, USA) through a Chip Cube-LC interface and nanospray ionization in positive mode. Data-dependent acquisition was used. For MS1, the mass range was 110–2000 *m*/*z* with a velocity of 4 spectra/s. The top 5 most intense precursor ions per cycle reaching 150 cps were selected for MS2 acquisition (50–2000 *m*/*z*) at a rate of 3 spectra/s. The active exclusion option was on, set to 2 spectra, and released after 0.25 min. A ramped collision energy was used with slope and offset values of 6 and 4, respectively. The equipment was externally calibrated before sample acquisition and every 24 h with an ESI-L low mix concentration tuning mix solution (Agilent Technologies, Inc., Santa Clara, CA, USA) to ensure a mass accuracy <5 ppm for MS1 and MS2 data.

#### 3.4.3. LC-MS/MS Data Processing

We followed a workflow previously described by our group [28]. In brief, raw LC-MS/MS datasets (.d) were converted to open-source (mzXML) and processed with open-access software and online platforms to perform two main tasks: (1) feature extraction and (2) annotation/identification of metabolites at the structural (metabolomics standard initiative [MSI] classification, levels 2 and 3) and chemical class (MSI classification, level 3) levels [64,65]. The overlapping patterns of the features and chemical classes identified among the plant’s anatomical parts were determined by UpSet plotting using the ComplexUpSet package based on the UpsetR package in R [63,66]. The molecular structures were generated by ChemDraw Professional software (version 16.0.1.4). The detailed processing parameters for all the steps are found in the supplemental experimental methods [28,65,66,67,68,69,70,71,72,73,74].

### 3.5. Chemical Space Comparison among FDA-Approved Drugs and P. brachystachyum Metabolites

To generate a visual representation of the chemical space among molecules, we utilized t-distributed stochastic neighbor embedding (t-SNE) as the dimensionality reduction method. Two sets of molecules were used for comparative purposes: (a) FDA-approved drugs retrieved from the ChEMBL database (https://www.ebi.ac.uk/chembl/g/#search_results/all/query=FDA, accessed and downloaded on 1 March 2023) and (b) *P. brachystachyum* metabolites putatively identified in this study. Compounds were identified by their SMILES, and duplicates were eliminated. The DataWarrior program (version 5.5.0) [75] was used for visual analysis with the following parameters selected: descriptor, FragFp; perplexity, 30; source dimensions, 50, and; interactions, 1000.

### 3.6. Antioxidant Capacity

#### 3.6.1. DPPH Radical Inhibition Assay

The antioxidant capacity of mistletoe methanolic extracts was determined using the methodology proposed by Brand–Williams et al. [76], with modifications. For the test, in a microplate of 96 wells, 10 μL of the extract was mixed at different concentrations, and 190 μL of the 200 µM DPPH reagent (2,2-difenyl-1-picrilhydrazyl) was added. The plate was incubated at room temperature for 30 min in darkness, and the absorbance was read at a wavelength of 540 nm in a microplate reader. Subsequently, the percentage inhibition of each concentration was obtained with the formula:

DPPH Inhibition (%) = ((Blank Absorbance − Extract Absorbance)/Blank Absorbance)) × 100

The equation of the line was calculated by clearing “x” and giving “y” a value of 50, and with the obtained data, the IC_50_ was obtained. The results obtained were expressed in DPPH IC_50_ of the leaf, stem, and aerial parts of *Phoradendron brachystachyum*.

x=(y−b)/m

#### 3.6.2. TEAC Assay

The antioxidant capacity of mistletoe methanolic extracts was determined as described by Thaipong et al. [77], with modifications. The ABTS was dissolved in distilled water at a concentration of 7.4 mM (the mother solution). The radical ABTS^•+^ was produced by mixing the ABTS mother solution with 2.6 mM (1:1 *v*/*v*) potassium persulfate and incubating the mixture in darkness at room temperature for 12–16 h before use. The solution with the radical was diluted in methanol until an absorbance of 0.70 ± 0.05 to 734 nm was reached. For the test, 10 μL of the extract was mixed with 190 μL of the reaction solution in a microplate, placed in incubation in darkness for 30 min, and the absorbance was read in a microplate reader at 734 nm. Subsequently, the percentage inhibition of each concentration was obtained with the formula:

ABTS Inhibition (%) = ((Blank Abs − Extract Abs)/Blank Absorbance)) × 100

where Blank Abs is the blank absorbance and Extract Abs is the extract absorbance. Also, the equation of the line was calculated by clearing “x” and giving “y” a value of 50, and with the obtained data, the IC_50_ was obtained. The results obtained were expressed in the ABTS IC_50_ of the leaf, stem, and aerial parts of *Phoradendron brachystachyum*.

x=(y−b)/m

#### 3.6.3. ORAC Assay

The ORAC assay was conducted as previously described by Gutiérrez-Grijalva, Angulo-Escalante, León-Félix, and Heredia [61], using fluorescein as the fluorescent probe, AAPH (2,2-azobis (2-amidino-propane) dihydrochloride) as a peroxyl radical generator, and Trolox as a standard. The reaction mixture contained 25 μL of mistletoe extract, 25 μL of 75 mM phosphate buffer (pH 7.4), 75 μL of 0.8 M AAPH, and 200 μL of 0.106 μM fluorescein. The samples, phosphate buffer, and fluorescein were pre-incubated at 37 °C for 15 min. The AAPH radical generator was added to start the reaction, and the fluorescence kinetics were monitored every 70 s for 70 min with a 485 nm excitation filter and a 580 nm emission filter (Synergy HT spectrophotometer). The values were calculated using a regression equation describing the relationship between the trolox concentration and the net area under the fluorescein decay curve, and a Trolox curve from 6.25 to 125 μmol TE/g was used to calculate the results, expressed as μmol of trolox equivalent per gram of sample (μmol TE/g), and the 75 mM phosphate buffer was used as a blank. Each sample was measured in triplicate (n = 3). Dilutions were prepared when needed.

#### 3.6.4. FRAP Assay

The FRAP assay was conducted according to Ali, Vahid, and Hawa [42], with modifications. Stock solutions included 400 mM acetate buffer, 30 mM TPTZ (2,4,6-tripyridyl-s triazine) solution in 0.1 mL of concentrated HCl, and 60 mM FeCl3 solution. Acetate buffer (10 mL), TPTZ (1 mL), and FeCl_3_ (1 mL) were mixed to obtain the FRAP reactive. A total of 30 μL of plant extracts were added, then 110 μL of the FRAP solution; the solution was mixed three times with the micropipette and kept for 4 min in the dark. The absorbance was measured at 630 nm using a spectrophotometer that had previously shaken the microplate for 1 min. From the calibration curve of the standard and the curve generated by the sample, the antioxidant capacity is obtained based on the reduction of ferric ions in the sample, expressed as the Mmol equivalent of Trolox per gram of *Phoradendron brachystachyum* (Mmol TE/g).

### 3.7. Total Phenolic Content

The total phenolic content of mistletoe extracts was determined by the Folin–Ciocalteu method, as indicated by the methodology of Swain and Hillis [78]. For the determination, in a Costar microplate^®^ of 96 wells, a 10 μL sample was mixed with 230 μL of distilled water and 10 μL of the Folin–Ciocalteu 2 N reagent and incubated for 3 min. Subsequently, 25 μL of Na_2_CO_3_ 4 N were added and incubated for 2 h in darkness; their absorbance was read at 725 nm in a Synergy HT microplate reader (Biotek, Inc., Winooski, VT, USA). The quantification of total phenolics was carried out by a standard curve of gallic acid, and the results were expressed in mg equivalents of gallic acid per g of extract (mg GAE/g sample).

### 3.8. Total Flavonoid Content

The total flavonoid content in the extracts of *P. brachystachyum* was determined by the colorimetric method reported by Ghasemi et al. [79] with some modifications. In a microplate, 10 μL of the sample was mixed with 250 μL of distilled water, 10 μL of AlCl_3_ (10% *w*/*v*), and 10 μL of potassium acetate (1 M). The solutions were incubated for 30 min in darkness, and the absorbance was read in a microplate reader at 415 nm. The quantification of total flavonoids was carried out using a quercetin curve, and the results were expressed as mg equivalents of quercetin per μg extract (mg QE/g sample) (n = 3).

### 3.9. Tannin Quantification

For the extraction of tannins, 100 mg of PVPP was weighed in an Eppendorf tube, to which 1 mL of distilled water and 1 mL of *P. brachystachyum* were added. A vortex was applied, and the mixture was allowed to incubate in refrigeration (4 °C) for 15 min. Then, the supernatant was stirred and centrifuged at 3500 rpm at 4 °C for 5 min. The supernatant was recovered, and the phenolic compound content (non-tannin compounds) was determined by the Folin–Ciocalteu assay.

#### 3.9.1. Total Tannin Content

The total tannins were obtained by difference; first, the content of phenolic compounds was determined by the Folin–Ciocalteu assay using a standard calibration curve of catechin, and later, to that value, the content of non-tannin phenolics was subtracted and expressed as milligrams of catechin equivalent per gram of sample, mg CE/g sample (n = 3).

TT = Phenolic compounds − non-tannin phenolics

#### 3.9.2. Condensed Tannins

The condensed tannins were determined by taking 20 μL of *P. brachystachyum* samples and depositing them in a 96-well microplate. A total of 200 μL of 0.1% DMCA solution was added and left to rest for 5 min at room temperature. Subsequently, its absorbance at 640 nm was measured with a standard catechin calibration curve, and the content was expressed as mg CE/g sample.

#### 3.9.3. Hydrolysable Tannins

Hydrolyzable tannins (TH) were obtained by subtracting the condensed tannin (CT) content from the total tannin content (TT).

TH = TT − TC

### 3.10. Pancreatic Lipase Inhibition Assay

The inhibitory activity of mistletoe extracts on pancreatic lipase was determined using the pancreatic spectroscopy method of Worsztynowicz et al. [80] with some modifications. In the trial, *p*-nitrophenyl palmitate (*p*NPP) was used as a substrate, which is hydrolyzed by the action of lipase to *p*-nitrophenol (*p*NP), a color agent that can be read at 410 nm. The total test consists of 1.8 mL of sodium phosphate buffer 0.05 M (pH 7.6 at a temperature of 37 °C) containing sodium cholate (1.15 mg/mL) and Arabic gum (0.55 mg/mL); *p*NPP was incubated at 37 °C with 0.2 mL in isopropanol (0.01 M) and 0.02 mL of a mistletoe extract at different concentrations (1, 2, 4, 8, 50, and 100 μg/mL). Then, 0.02 mL of the swine lipase enzyme solution (50 mg/mL) was added to sodium phosphate buffer (0.05 M) for 30 min to initiate the colorimetric reaction. The released *p*-nitrophenol was read at 410 nm. Therefore, the control reaction (without mistletoe extracts) represents a blank (100% enzymatic activity). Subsequently, the inhibition percentage of each concentration was obtained with the formula ((Blank Absorbance − Sample Absorbance)/Blank Absorbance)) × 100; the equation of the line was calculated by clearing “x” and giving “y” a value of 50; with the obtained data, the IC_50_ was obtained. The results were expressed in the pancreatic lipase IC50 of the leaf, stem, and aerial parts of *Phoradendron brachystachyum*.

x=(y−b)/m

### 3.11. α-Glucosidase Inhibition Assay

The inhibitory activity of α-glucosidase was determined according to a modified assay from the Worthington Enzyme Manual as reported in the literature [37]. In 96-well microplates, 50 μL of methanolic extract of *Phoradendron brachystachyum* were incubated for 10 min at 37 °C with 100 μL of α-glucosidase of *Saccharomyces cerevisiae* (0.6 U mL^−1^) in phosphate buffer (0.1 M, pH 6.9). Then, 50 μL of 3 mM p-nitrophenyl-α-glucopyranoside was added to the pH 6.9 phosphate buffer (pNPG), and the mixture was incubated again for 12 min at 37 °C. Enzyme activity was determined by measuring the release of ρ-nitrophenol from the ρNPG substrate. Absorbance at 405 nm was measured with a 96-well microplate reader (Synergy HT, Bio-Tek Instruments, Inc., Winooski, VT, USA). In addition, 50 μL of 1 mM acarbose was used as a control).

Subsequently, the percentage inhibition of each concentration was obtained with the formula:

α-Glucosidase inhibition (%) = ((Blank Abs − Extract Abs)/Blank Absorbance)) × 100

where Blank Abs is the blank absorbance and Extract Abs is the extract absorbance. Also, the equation of the line was calculated by clearing “x” and giving “y” a value of 50; with the obtained data, the IC_50_ was obtained. The results obtained were expressed in α-Glucosidase IC_50_ of leaf, stem, and aerial parts of *Phoradendron brachystachyum*.

x=(y−b)/m

## 4. Conclusions

A total of 438 metabolites were identified in *P. brachystachyum*, of which some of the most important for the ethnopharmacological properties of American mistletoe are flavonoids, which were found in all plant parts. Interestingly, the highest number of unique metabolites was found in leaf extracts. Moreover, among the most important compounds found in mistletoe extracts were ellagic acid, quercetin, quercetin glycosides, kaempferol glycosides, and the tannins epicatechin gallate, procyanidin B1, 2′-O-galloylquercitrin, and 1,2,3-Tri-O-galloyl-beta-D-glucose. Also, leaf extracts showed higher values in antioxidant capacity and total phenolic and flavonoid content. Leaf extracts of *Phoradendron* also showed the best inhibitory activity against α-glucosidase and pancreatic lipase. Moreover, further studies will focus on the quantitative analysis of the identified flavonoids and tannins and their bioaccessibility and bioavailability.

## Figures and Tables

**Table 1:** Phytochemical preliminary screening of *Phoradendron brachystachyum*.

Phytochemical	Assay/Reagent	Solvent	Leaf	Stem
Tannins	Ferric chloride	Hexane	−	−
		Methanol	+++	+++
		Water	+++	+++
Flavonoids	Shinoda	Hexane	+	−
		Methanol	+	+
		Water	++	++
Coumarins	Baljet	Hexane	+	+
		Methanol	+++	+++
		Water	++	++
Saponins	Foam	Hexane	−	−
		Methanol	−	−
		Water	+	+
Terpenes	Liberman-Bouchard	Hexane	+	−
		Methanol	+	−
		Water	−	−
	Salkowiski	Hexane	+	+
		Methanol	+	+
		Water	+	+
Alkaloids	Dragendorff	Hexane	−	−
		Methanol	−	−
		Water	−	−
	Mayer	Hexane	−	−
		Methanol	+	+
		Water	−	−
	Wagner	Hexane	+	+
		Methanol	+	+
		Water	−	−

(+): low presence (color change), (++): medium presence (color change and low precipitate formation), (+++) high presence (Intense color change and high precipitate formation), (−): negative, the experiment was done by triplicate of each solvent and sample.

**Table 2:** Predicted passive absorption of some compounds found *Phoradendron brachystachyum*.

Molecule	Molecular Weight	H Bond Donors	H Bond Acceptors	Log P	Lipinski’s Rule of 5
Naringenin	272.25 g/mol	3	5	1.84	YES
Phloretin	274.27 g/mol	4	5	1.93	YES
Sakuranetin	286.28 g/mol	0	4	2.25	YES
Quercetin	302.24 g/mol	5	7	1.23	YES
Quercetin 3-beta-D-galactopyran	464.38 g/mol	8	12	−0.25	NO
6-[3-[(3,4-dimethoxyphenyl)methyl]-4-methoxy-2-(methoxymethyl)butyl]-4-methoxy-1,3-benzodioxole	432.51 g/mol	0	7	3.89	YES
2-(3,4-dihydroxyphenyl)-5,7-dihydroxy-3-[(2S,3R,4S,5S,6R)-3,4,5-trihydroxy-6-[[(2S,3R,4S,5R)-3,4,5-trihydroxyoxan-2-yl]oxymethyl]oxan-2-yl]oxychromen-4-one	596.49 g/mol	10	16	−1.61	NO
Afzelin	432.38 g/mol	6	10	0.6	YES
Guajaverin	434.35 g/mol	7	11	0	NO
3-[(2S,3R,4S,5S,6R)-4,5-dihydroxy-6-(hydroxymethyl)-3-[(2S,3R,4S,5R)-3,4,5-trihydroxyoxan-2-yl]oxyoxan-2-yl]oxy-2-(3,4-dihydroxyphenyl)-5-hydroxy-7-methoxychromen-4-one	610.52 g/mol	9	16	−1.1	NO
Quercetin-3-O-alpha-L-rhamnopyranoside	448.38 g/mol	7	11	0.16	NO
(2S,3S,4S,5R,6S)-3,4,5-trihydroxy-6-[5-hydroxy-2-(4-hydroxyphenyl)-6-methoxy-4-oxochromen-7-yl]oxyoxane-2-carboxylic acid	476.39 g/mol	6	12	0.25	NO
5,8-dihydroxy-2-(4-hydroxyphenyl)-7-methoxy-3-[(2S,3R,4R,5R,6S)-3,4,5-trihydroxy-6-methyloxan-2-yl]oxychromen-4-one	462.40 g/mol	6	11	0.71	NO
(+) Catechin	290.27 g/mol	5	6	0.85	YES
1-(4-methoxyphenyl)ethanone	150.17 g/mol	0	2	1.83	YES
5-hydroxy-2-(4-hydroxyphenyl)-3-[(2S,3R,4S,5S,6R)-3,4,5-trihydroxy-6-(hydroxymethyl)oxan-2-yl]oxy-7-[(2S,3R,4R,5R,6S)-3,4,5-trihydroxy-6-methyloxan-2-yl]oxychromen-4-one	594.52 g/mol	9	15	−1.12	NO
Ellagic acid	302.19 g/mol	4	8	1	YES
5,7-dihydroxy-2-(4-hydroxyphenyl)-3-[(2S,3R,4S,5S,6R)-3,4,5-trihydroxy-6-[[(2S,3R,4S,5S)-3,4,5-trihydroxyoxan-2-yl]oxymethyl]oxan-2-yl]oxychromen-4-one	580.49 g/mol	9	15	1.39	NO
isorhamnetin-3-O-glucoside	478.40 g/mol	7	12	−0.15	NO
Spiraeoside	464.38 g/mol	8	12	−0.19	NO
Isorhamnetin 3-galactoside	478.40 g/mol	7	12	−0.15	NO
5-hydroxy-2-(4-hydroxyphenyl)-3-[(2S,3R,4S,5S,6R)-3,4,5-trihydroxy-6-(hydroxymethyl)oxan-2-yl]oxy-7-[(2S,3R,4R,5R,6S)-3,4,5-trihydroxy-6-methyloxan-2-yl]oxychromen-4-one	594.52 g/mol	9	15	−1.12	NO
Kaempferol 3-alpha-L-arabinopyranoside	418.35 g/mol	6	10	0.24	YES
[(2S,3R,4R,5S,6S)-2-[2-(3,4-dihydroxyphenyl)-5,7-dihydroxy-4-oxochromen-3-yl]oxy-3,5-dihydroxy-6-methyloxan-4-yl] 3,4,5-trihydroxybenzoate	600.48 g/mol	9	15	0.74	NO
3-[(2S,3R,4S,5S,6R)-4,5-dihydroxy-6-(hydroxymethyl)-3-[(2S,3R,4S,5R)-3,4,5-trihydroxyoxan-2-yl]oxyoxan-2-yl]oxy-5,7-dihydroxy-2-(4-hydroxyphenyl)chromen-4-one	580.49 g/mol	9	15	−1.42	NO
Naringin	580.53 g/mol	8	14	−0.79	NO
Isoquercitin	464.38 g/mol	8	12	−0.25	NO
5-hydroxy-3-[(2S,3R,4R,5S)-3-hydroxy-5-(hydroxymethyl)-4-[(2S,3R,4S,5S,6R)-3,4,5-trihydroxy-6-(hydroxymethyl)oxan-2-yl]oxyoxolan-2-yl]oxy-2-(4-hydroxyphenyl)-7-[(2S,3R,4R,5R,6S)-3,4,5-trihydroxy-6-methyloxan-2-yl]oxychromen-4-one	726.63 g/mol	11	19	−2.25	NO
5,8-dihydroxy-2-(4-hydroxyphenyl)-7-methoxy-3-[(2S,3R,4R,5R,6S)-3,4,5-trihydroxy-6-methyloxan-2-yl]oxychromen-4-one	462.40 g/mol	6	11	0.71	NO
5-hydroxy-2-(4-hydroxyphenyl)-7-(3,4,5-trihydroxy-6-methyloxan-2-yl)oxy-3-(3,4,5-trihydroxyoxan-2-yl)oxychromen-4-one	564.49 g/mol	8	14	−0.69	NO
5,7-dihydroxy-2-(4-hydroxyphenyl)-2,3-dihydro-4H-chromen-4-one	272.25 g/mol	3	5	1.84	YES
Vitamin-P	610.52 g/mol	10	16	−1.29	NO
Secoisolariciresinol	362.42 g/mol	4	6	2.5	YES
Epicatechin gallate	442.37 g/mol	7	10	1.25	YES
Rhamnetin 3-galactoside	478.40 g/mol	7	12	0.06	NO
Hyperoside	464.38 g/mol	8	12	−0.25	NO
2-(3,4-dihydroxyphenyl)-5,7-dihydroxy-3-[(2S,3R,4S,5S)-4-hydroxy-5-(hydroxymethyl)-3-[(2S,3R,4S,5R)-3,4,5-trihydroxyoxan-2-yl]oxyoxolan-2-yl]oxychromen-4-one	566.46 g/mol	9	15	−1	NO
Quercetin 3-O-robinobioside	610.52 g/mol	10	16	−1.29	NO
Tamarixetin	316.26 g/mol	4	7	1.85	YES
Procyanidin B1	578.52 g/mol	10	12	1.53	NO
Kaempferol 3-O-arabinoside	418.35 g/mol	6	10	0.24	YES
Quercetin-3-O-xyloside	434.35 g/mol	7	11	0	NO
Quercetin 3,7-dirhamnoside	594.52 g/mol	9	15	−0.98	NO
Kaempferol	286.24 g/mol	4	6	1.58	YES
2-(3,4-dihydroxyphenyl)-5,8-dihydroxy-7-methoxy-3-[(2S,3R,4R,5R,6S)-3,4,5-trihydroxy-6-methyloxan-2-yl]oxychromen-4-one	478.40 g/mol	7	12	0.28	NO
Avicularin	434.35 g/mol	7	11	0.19	NO
2’-O-Galloylquercitrin	600.48 g/mol	9	15	0.77	NO
Luteolin 4’-O-glucoside	448.38 g/mol	7	11	0.2	NO
1,2,3-Tri-O-galloyl-beta-D-glucose	636.47 g/mol	11	18	−0.52	NO

Predicted passive absorption of molecules found in the Phoradendron brachystachyum sample extracts indicated with YES or NO by Lipinski’s rule of 5.

**Table 3:** Total phenolic and flavonoid content of hydrophilic extracts from leaf, stem, and aerial parts of mistletoe (*P. brachystachyum*).

Part	Total Phenolic Content (mg GAE/g Sample)	Total Flavonoids (mg QE/g Sample)
Leaf	142.11 ± 10.28 ^a^	22.67 ± 1.06 ^a^
Stem	73.78 ± 6.67 ^c^	15.64 ± 0.43 ^c^
Aerial parts	123.62 ± 9.54 ^b^	19.37 ± 0.71 ^b^

GAE: Gallic acid equivalents, QE: Quercetin equivalents. Data displayed as means ± standard deviation of 3 replicas with 3 repetitions per replica. Different letters in the same column indicate significant differences by Tukey test (*p* < 0.05).

**Table 4:** Total, condensed, and hydrolyzable tannin content of hydrophilic extracts from leaf, stem, and aerial parts of mistletoe (*P. brachystachyum*) (mg CE/g sample).

Part	Total Tannins	Condensed Tannins	Hydrolyzable Tannins
Leaf	142.59 ± 4.94 ^a^	57.04 ± 5.02 ^a^	85.54 ± 9.91 ^a^
Stem	85.71 ± 3.77 ^b^	33.82 ± 0.96 ^b^	51.88 ± 3.77 ^b^
Aerial parts	137.65 ± 1.43 ^a^	52.84 ± 6.69 ^a^	84.81 ± 5.27 ^a^

CE: Catechin equivalents. Data displayed as means ± standard deviation of 3 replicates with 3 repetitions per replicate. Different letters in the same column indicate significant differences by Tukey test (*p* < 0.05).

**Table 5:** Antioxidant capacity of mistletoe (*P. brachystachyum*) hydrophilic extracts by the DPPH, TEAC, ORAC, and FRAP methods.

Part	DPPH (IC_50_, μg/mL)	TEAC (IC_50_, μg/mL)	ORAC Values (μmol TE/g Sample)	FRAP Values (Mmol TE/g Sample)
Leaf	14.09 ± 0.05 ^ab^	6.69 ± 0.04 ^c^	1150.93 ± 28.43 ^a^	4006.30 ± 306.76 ^a^
Stem	14.97 ± 0.48 ^a^	7.99 ± 0.03 ^b^	1017.92 ± 23.09 ^b^	2780.19 ± 135.40 ^c^
Aerial parts	13.57 ± 0.57 ^b^	8.32 ± 0.16 ^a^	1163.29 ± 38.21 ^a^	3581.16 ± 197.08 ^b^
Ascorbic acid	170.21	85.39	-	-

DPPH: 2,2-diphenyl-1-picrylhydrazyl, TEAC: Trolox equivalent antioxidant capacity, ORAC: Oxygen radical absorbance capacity, FRAP: Ferric reducing antioxidant potential; IC_50_: concentration of the extract that inhibits 50% of the radical activity. Data displayed as means ± standard deviation of 3 replicates with 3 repetitions. Different letters in the same column indicate significant differences by Tukey test (*p* < 0.05).

**Table 6:** Inhibitory percentage of hydrophilic extracts from leaf, stem, and aerial parts of mistletoe (*P. brachystachyum*) against α-glucosidase and pancreatic lipase.

Sample	Concentration	Inhibition α-Glucosidase (%)	Concentration	Inhibition Pancreatic Lipase (%)
Leaf	15 μg/mL	32.73 ± 2.55 ^a^	50 μg/mL	71.44 ± 1.05 ^a^
Stem	15 μg/mL	29.36 ± 1.67 ^b^	50 μg/mL	49.11 ± 1.27 ^b^
Aerial parts	15 μg/mL	24.13 ± 1.36 ^c^	50 μg/mL	51.71 ± 0.94 ^b^
Acarbose	1 mM	58.33 ± 0.86		
Orlistat			60 μg/mL	72.38 ± 0.80

Data displayed as means ± standard deviation of 3 replicas with 3 repetitions per replica. Different letters in the same column indicate significant differences by Tukey test.

## Data Availability

The raw LC-MS2 datasets have been deposited on the GNPS/MassIVE public repository under the accession number MSV000091508. The parameters and data for GNPS-derived analysis are available at the following links: (a) classical molecular networking/spectral matching, https://gnps.ucsd.edu/ProteoSAFe/status.jsp?task=48e26aaaf99b49d6a0632017ced8aaad; (accessed on 6 April 2023) (b) Moldiscovery, https://gnps.ucsd.edu/ProteoSAFe/status.jsp?task=209b5df16ac24bce8e0be94b90b024c4 (accessed on 6 April 2023); (c) dereplicator+, https://gnps.ucsd.edu/ProteoSAFe/status.jsp?task=198374a72f5449d9a4b72629f2943223 (accessed on 6 April 2023).

## Supplementary Materials

The following supporting information can be downloaded at: https://www.mdpi.com/article/10.3390/plants12142729/s1. Table S1. List of putatively annotated metabolites (SIRIUS 4.9.12) in mistletoe by anatomical site.
