# Supplementary material for: Metabolomic Characterization of Phoradendron brachystachyum Mistletoe and In-Silico and In-Vitro Investigation of Its Therapeutic Potential in Metabolic Disorders
Source: Plants (Basel). 2023 Jul 22;12(14):2729. doi: 10.3390/plants12142729 (PMC10383180; doi:10.3390/plants12142729)
Supplement: Supplementary file 1 [file plants-12-02729-s001.zip › 04 Supplementary Table S1_IdentifiedCompounds_Toji_20march2023.pdf]

Table S1. List of putatively annotated metabolites (SIRIUS 4.9.12) in mistletoe by anatomical site.

ID	Name	Annotation Tool	Superclass	Class	Subclass	Molecular Formula	Adduct	Theoretical mass	Experimental mass	Mass accuracy (ppm)	Retention time (min)	Location
1	2-[2-hexyl-2-[[3,4,5-trihydroxy-6-(hydroxymethyl)oxan-2-yl]oxymethyl]octoxy]-6-(hydroxymethyl)oxane-3,4,5-triol	CSI:FingerID	Lipids and lipid-like molecules	Fatty Acyls	Fatty acyl glycosides	C27H52O12	[M + H]+	569.3531	569.3488	-7.4461	18.2127	Aerial Leaf
2	9-{1-[3,5-bis(hydroxymethyl)-4-methyloxolan-2-yl]-1,2-dihydroxypropan-2-yl}-8,23-dimethyl-17-phenyl-13,19-dioxapentacyclo[18.3.1.0?,??.0?,??.0?,??]tetracos-3-en-15-yn-2-one	CSI:FingerID	Lipids and lipid-like molecules	Steroids and steroid derivatives	Ecdysteroids	C40H54O8	[M - H2O + H]+	645.3791	645.3808	2.5934	24.0667	Aerial Leaf
3	5-[[4-[4-[[2,6-diamino-3-[(4-sulfophenyl)diazenyl]phenyl]diazenyl]-3-methylphenyl]-2-methylphenyl]diazenyl]-2-hydroxybenzoic acid	CSI:FingerID	Benzenoids	Benzene and substituted derivatives	Biphenyls and derivatives	C33H28N8O6S	[M + K]+	703.149	703.15	1.5376	16.1511	Aerial Leaf

4	(2-(4-(2-(5-amino-1H-pyrrol-2-yl)ethoxy)phenyl)-7-((6-carboxy-3,4,5-trihydroxytetrahydro-2H-pyran-2-yl)oxy)-6-((3-ethyl-5-hydroxybenzyl)oxy)-5-hydroxy-4H-chromen-4-ylidene)oxonium	CSI:FingerID	Phenylpropanoids and polyketides	Flavonoids	Flavonoid glycosides	C36H36N2O13	[M + Na]+	727.2115	727.2071	-6.0539	12.0242	Aerial Leaf
5	Nicotiflorin	GNPS	Phenylpropanoids and polyketides	Flavonoids	Flavonoid glycosides	C27H30O15	[M + H]+	595.1657	595.1656	-0.0786	12.831	Aerial Leaf
6	PA(14:1(9Z)/18:4(6Z,9Z,12Z,15Z))	MolDiscovery	Lipids and lipid-like molecules	Glycerophospholipids	Glycerophosphates	C35H59O8P	[M + H]+	639.402	639.396	-9.3142	18.5547	Aerial Leaf
7	2-[[2-[[2-[(2-amino-3-phenylpropanoyl)amino]-3-methylbutanoyl]amino]-3-methylbutanoyl]amino]-3-(4-hydroxyphenyl)propanoic acid	CSI:FingerID	Organic acids and derivatives	Carboxylic acids and derivatives	Amino acids, peptides, and analogues	C28H38N4O6	[M - H2O + H]+	509.2764	509.2741	-4.4688	24.0333	Aerial Stem
8	1,3-dimethylxanthosine	CSI:FingerID	Nucleosides, nucleotides, and analogues	Purine nucleosides		C12H16N4O6	[M - H2O + H]+	295.1042	295.1039	-1.1895	19.8284	Aerial Stem Leaf

9	phytosphingosine	CSI:FingerID	Organic nitrogen compounds	Organonitrogen compounds	Amines	C18H39NO3	[M - H2O + H]+	300.2903	300.2886	-5.6592	19.5812	Aerial Stem Leaf
10	(2S)-2-hydroxy-3-(8-hydroxy-6-methoxy-1-oxoisochromen-3-yl)propanoic acid	CSI:FingerID	Phenylpropanoids and polyketides	Isocoumarins and derivatives		C13H12O7	[M + Na]+	303.0481	303.0494	4.3461	14.2716	Aerial Stem Leaf
11	N-butyl-4-(1,1,1,3,3,3-hexafluoropropan-2-yloxy)-6-piperazin-1-yl-1,3,5-triazin-2-amine	CSI:FingerID	Organoheterocyclic compounds	Diazinanes	Piperazines	C14H20F6N6O	[M + H]+	403.1675	403.1647	-6.9887	18.3736	Aerial Stem Leaf
12	Cibotiumbaroside A	CSI:FingerID	Organic oxygen compounds	Organooxygen compounds	Carbohydrates and carbohydrate conjugates	C18H20O11	[M + Na]+	435.0903	435.09	-0.7117	13.0876	Aerial Stem Leaf
13	Benzoic acid, 3,5-dinitro-, (1,2-dihydro-2-oxo-1-(1-piperidinylmethyl)-3H-indol-3-ylidene)hydrazide	CSI:FingerID	Organoheterocyclic compounds	Indoles and derivatives	N-alkylindoles	C21H20N6O6	[M + H]+	453.1516	453.1555	8.5545	16.4257	Aerial Stem Leaf
14	2-Tetradecanoylaminohexanol-1-phosphocholine	CSI:FingerID	Organic nitrogen compounds	Organonitrogen compounds	Quaternary ammonium salts	C25H53N2O5P	[M + H]+	493.3764	493.3727	-7.5755	23.5303	Aerial Stem Leaf
15	2-[(4-carboxylato-3,3-dimethylbutanamido)methyl]-5-[6-(pentan-3-yl)-	CSI:FingerID	Organoheterocyclic compounds	Diazines	Pyrimidines and pyrimidine derivatives	C29H41N5O3	[M + H3N + H]+	525.3553	525.3585	6.0708	17.515	Aerial Stem Leaf

	2-(pyridin-4-yl)pyrimidin-4-yl]-1-azabicyclo[2.2.2]octan-1-ium											
16	2-(aminomethyl)-6-[(4,6-diamino-3-{[3,5-dihydroxy-6-(hydroxymethyl)-4-(methylamino)oxan-2-yl]oxy}-2-hydroxycyclohexyl)oxy]oxane-3,4,5-triol	CSI:FingerID	Organic oxygen compounds	Organooxygen compounds	Carbohydrates and carbohydrate conjugates	C19H38N4O11	[M + K]+	537.2174	537.213	-8.259	19.3432	Aerial Stem Leaf
17	Ser-Thr-Gly-Gly-Tyr-Gly	CSI:FingerID	Organic acids and derivatives	Carboxylic acids and derivatives	Amino acids, peptides, and analogues	C22H32N6O10	[M + Na]+	563.2078	563.2088	1.8913	17.1325	Aerial Stem Leaf
18	2-[(1-Hydroxy-6-oxo-2-cyclohexenyl)carbonyloxymethyl]phenyl 2-O-(4-hydroxy-trans-cinnamoyl)-beta-D-glucopyranoside	CSI:FingerID	Organic oxygen compounds	Organooxygen compounds	Carbohydrates and carbohydrate conjugates	C29H30O12	[M + Na]+	593.1635	593.1663	4.7434	15.0668	Aerial Stem Leaf
19	Fomidacillin	CSI:FingerID	Organic acids and derivatives	Carboxylic acids and derivatives	Amino acids, peptides, and analogues	C24H28N6O10S	[M + H]+	593.166	593.1661	0.1932	12.3653	Aerial Stem Leaf
20	UDPglucose	CSI:FingerID	Nucleosides, nucleotides	Pyrimidine nucleotides	Pyrimidine nucleotide sugars	C15H24N2O17P2	[M + K]+	605.0187	605.0187	-0.1239	11.7225	Aerial Stem Leaf

			, and analogues									
21	N-[2-[[3-[[1-(cyclohexylmethyl)piperidin-3-yl]methylamino]-3-oxopropyl]amino]-2-oxoethyl]-3,3,3-triphenylpropanamide	CSI:FingerID	Organic acids and derivatives	Peptidomimetics	Hybrid peptides	C39H50N4O3	[M + H]+	623.3955	623.3994	6.3164	24.0944	Aerial Stem Leaf
22	Sporidesmolide III	CSI:FingerID	Organic acids and derivatives	Peptidomimetics	Depsipeptides	C32H56N4O8	[M + H]+	625.417	625.4146	-3.7853	23.2018	Aerial Stem Leaf
23	N-[(2R,3S)-5-[(2R)-1-hydroxypropan-2-yl]-2-[[(4-methoxyphenyl)sulfonyl-methylamino]methyl]-3-methyl-6-oxo-2,3,4,7-tetrahydro-1,5-benzoxazonin-9-yl]-4-methoxybenzenesulfonamide	CSI:FingerID	Benzenoids	Benzene and substituted derivatives	Sulfanilides	C31H39N3O9S2	[M + H]+	662.22	662.2264	9.7619	14.355	Aerial Stem Leaf
24	Cyclo(grgdspa)	CSI:FingerID	Organic acids and derivatives	Peptidomimetics	Hybrid peptides	C24H38N10O12	[M + Na]+	681.2568	681.2524	-6.5781	15.9421	Aerial Stem Leaf
25	[(2R,3S,4S,5R,6R)-5-acetyloxy-6-[[(2R,3S,4S,5R,6S)-6-[2-(3,4-dihydroxyphenyl)-5,7-	CSI:FingerID	Phenylpropanoids and polyketides	Flavonoids	Flavonoid glycosides	C31H34O19	[M + Na]+	733.1592	733.1609	2.2612	15.2504	Aerial Stem Leaf

	dihydroxy-4-oxochromen-3-yl]oxy-3,4,5-trihydroxyoxan-2-yl]methoxy]-3,4-dihydroxyoxan-2-yl]methyl acetate											
26	Multiroside	CSI:FingerID	Lipids and lipid-like molecules	Prenol lipids	Terpene glycosides	C31H42O19	[M + Na]+	741.2218	741.2175	-5.8098	17.3166	Aerial Stem Leaf
27	4-[(6-{[2-(3,4-dihydroxyphenyl)-5-hydroxy-4-oxo-7-{[3,4,5-trihydroxy-6-(hydroxymethyl)oxan-2-yl]oxy}-4H-chromen-3-yl]oxy}-3,4,5-trihydroxyoxan-2-yl)methoxy]-3-oxobutanoic acid	CSI:FingerID	Phenylpropanoids and polyketides	Flavonoids	Flavonoid glycosides	C31H34O20	[M + Na]+	749.1541	749.1561	2.7135	14.2939	Aerial Stem Leaf
28	(3S)-4-amino-3-[[(2S)-3-(4-hydroxyphenyl)-2-[[(2S)-3-(1H-indol-3-yl)-2-[[(2E)-2-[2-[(2S,3S,4S,5S,6R)-3,4,5-trihydroxy-6-(hydroxymethyl)oxan-2-yl]oxyethoxyimino]acetyl]amino	CSI:FingerID	Organic acids and derivatives	Carboxylic acids and derivatives	Amino acids, peptides, and analogues	C34H42N6O14	[M + H]+	759.2831	759.2845	1.8827	17.9242	Aerial Stem Leaf

	]propanoyl]amino]propanoyl]amino]-4-oxobutanoic acid											
29	6''-[2-(2-hydroxyethoxy)phenyl]-3'',4''-diphenyl-8''-(4-pyrimidin-2-ylpiperazine-1-carbonyl)spiro[1H-indole-3,7''-4,6,8,8a-tetrahydro-3H-pyrrolo[2,1-c][1,4]oxazine]-1'',2-dione	CSI:FingerID	Phenylpropanoids and polyketides	Stilbenes		C43H40N6O6	[M + Na]+	759.2907	759.2842	-8.5943	17.4034	Aerial Stem Leaf
30	Bis(benzylpenicilloyl)-1,6-diaminohexane	CSI:FingerID	Organic acids and derivatives	Carboxylic acids and derivatives	Amino acids, peptides, and analogues	C38H48N6O6S2	[M + Na]+	771.2974	771.2991	2.1248	17.5522	Aerial Stem Leaf
31	8,8'-Methylenebis[3,3',4',5,7-pentahydroxyflavan]	Dereplicator	Phenylpropanoids and polyketides	Flavonoids	Flavans	C31H28O12	[M + H]+	593.1653	593.166	1.22	14.9915	Aerial Stem Leaf
32	Alismol	GNPS	Lipids and lipid-like molecules	Prenol lipids	Sesquiterpenoids	C15H24O	[M - H2O + H]+	203.18	203.1781	-9.0821	23.5371	Aerial Stem Leaf
33	Naringenin	GNPS	Phenylpropanoids and polyketides	Flavonoids	Flavans	C15H12O5	[M + H]+	273.0757	273.0757	0.1238	15.3355	Aerial Stem Leaf
34	Phloretin	GNPS	Phenylpropanoids and	Linear 1,3-diarylpropanoids	Chalcones and	C15H14O5	[M + H]+	275.0913	275.0911	-0.8254	14.646	Aerial Stem Leaf

			polyketides		dihydrochalcones							
35	Kaempferol	GNPS	Phenylpropanoids and polyketides	Flavonoids	Flavones	C15H10O6	[M + H]+	287.0549	287.0552	0.8116	12.412	Aerial Stem Leaf
36	(-)-Epicatechin	GNPS	Phenylpropanoids and polyketides	Flavonoids	Flavans	C15H14O6	[M + H]+	291.0862	291.0855	-2.6032	5.5984	Aerial Stem Leaf
37	Peonidin-3-O-alpha-arabinoside	GNPS	Phenylpropanoids and polyketides	Flavonoids	Flavonoid glycosides	C21H20O10	[M + H]+	433.1128	433.1145	3.7495	16.4505	Aerial Stem Leaf
38	Quercitrin	GNPS	Phenylpropanoids and polyketides	Flavonoids	Flavonoid glycosides	C21H20O11	[M + H]+	449.1078	449.107	-1.7037	13.6848	Aerial Stem Leaf
39	Astragalin	GNPS	Phenylpropanoids and polyketides	Flavonoids	Flavonoid glycosides	C21H20O11	[M + H]+	449.1078	449.1067	-2.3185	13.1248	Aerial Stem Leaf
40	Isorhamnetin 3-galactoside	GNPS	Phenylpropanoids and polyketides	Flavonoids	Flavonoid glycosides	C22H22O12	[M + H]+	479.1183	479.1179	-0.9063	13.8285	Aerial Stem Leaf
41	Quercetin 3-xylosyl-(1->2)-alpha-L-arabinofuranoside	GNPS	Phenylpropanoids and polyketides	Flavonoids	Flavonoid glycosides	C25H26O15	[M + H]+	567.1344	567.1346	0.4681	12.3405	Aerial Stem Leaf

42	Leucoside	GNPS	Phenylpropanoids and polyketides	Flavonoids	Flavonoid glycosides	C26H28O15	[M + H]+	581.15	581.1512	1.9924	12.412	Aerial Stem Leaf
43	Peltatoside	GNPS	Phenylpropanoids and polyketides	Flavonoids	Flavonoid glycosides	C26H28O16	[M + H]+	597.1449	597.1448	-0.2062	11.4279	Aerial Stem Leaf
44	3-[(2S,3R,4S,5S,6R)-4,5-dihydroxy-6-(hydroxymethyl)-3-[(2S,3R,4S,5R)-3,4,5-trihydroxyoxan-2-yl]oxyoxan-2-yl]oxy-2-(3,4-dihydroxyphenyl)-5-hydroxy-7-methoxychromen-4-one	GNPS	Phenylpropanoids and polyketides	Flavonoids	Flavonoid glycosides	C27H30O16	[M + H]+	611.1606	611.1601	-0.8647	12.8588	Aerial Stem Leaf
45	3-{6-[((6S,2R,3R,4R,5R)-3,4,5-trihydroxy-6-methyl(2H-3,4,5,6-tetrahydropyran-2 -yloxy))methyl](2S,4S,5S,3R,6R)-3,4,5-trihydroxy(2H-3,4,5,6-	GNPS	Phenylpropanoids and polyketides	Flavonoids	Flavonoid glycosides	C27H30O16	[M + H]+	611.1606	611.1596	-1.6544	12.2554	Aerial Stem Leaf

	tetrahydropyran -2- yloxy)}-2-(2,3-dihydroxyphenyl)-5,7-dihydroxychromen-4-one											
46	Epigallocatechin -3-O-p-hydroxybenzoate-(4b-8)-epigallocatechin	MolDiscovery	Phenylpropanoids and polyketides	Flavonoids	Biflavonoids and polyflavonoids	C37H30O16	[M + H]+	731.1606	731.16	-0.7999	13.4892	Aerial Stem Leaf
47	Quercetin-3-(2'''-galloylglucosyl)-(1-2)-alpha-L-arabinofuranoside	MolDiscovery	Phenylpropanoids and polyketides	Flavonoids	Flavonoid glycosides	C33H32O20	[M + H]+	749.1559	749.156	0.1423	14.227	Aerial Stem Leaf
48	3,3',4,4',5,5',9-Heptahydroxy-7,9'-epoxylignan-3,3',5,5'-tetra-methyl ether, 9-O-[4-hydroxy-3-methoxy-E-cinnamoyl-(6)-D-glucopyranoside]	MolDiscovery	Lignans, neolignans and related compounds	Lignan glycosides		C38H46O16	[M + H]+	759.2858	759.286	0.2826	16.6707	Aerial Stem Leaf
49	Acanthoside D	MolDiscovery	Lignans, neolignans and related compounds	Lignan glycosides		C36H50O18	[M + H]+	771.3069	771.3	-8.9651	17.8657	Aerial Stem Leaf
50	Dolabriproanthocyanidin	MolDiscovery	Phenylpropanoids and	Flavonoids	Biflavonoids and polyflavonoids	C45H38O18	[M + H]+	867.213	867.21	-3.4759	10.5491	Aerial Stem Leaf

			polyketides									
51	Octa-acetyl-listvenol	MolDiscovery	Phenylpropanoids and polyketides	Diarylheptanoids	Linear diarylheptanoids	C46H40O18	[M + H]+	881.2287	881.227	-1.8888	13.8829	Aerial Stem Leaf
52	Epigallocatechin-3-O-p-hydroxybenzoate-(4b-8)-epigallocatechin-3-O-gallate	MolDiscovery	Phenylpropanoids and polyketides	Flavonoids	Biflavonoids and polyflavonoids	C44H34O20	[M + H]+	883.1715	883.174	2.7815	15.4014	Aerial Stem Leaf
53	Epicatechin-(4b-8)-epicatechin, 3-O-gallate-(4b-8)-catechin	MolDiscovery	Phenylpropanoids and polyketides	Flavonoids	Biflavonoids and polyflavonoids	C52H42O22	[M + H]+	1019.224	1019.22	-3.8981	12.3919	Aerial Stem Leaf
54	Camelliatannin B	MolDiscovery	Phenylpropanoids and polyketides	Tannins	Complex tannins	C49H36O27	[M + H]+	1057.1516	1057.15	-1.5096	12.5426	Aerial Stem Leaf
55	3,3',4',5,7-Pentahydroxyflavan-(4-8)-3,3',4',5,7-pentahydroxyflavan-(4-6)-3,3',4',5,7-pentahydroxyflavan-(4-8)-3,3',4',5,7-pentahydroxyflavan	MolDiscovery	Phenylpropanoids and polyketides	Flavonoids	Biflavonoids and polyflavonoids	C60H50O24	[M + H]+	1155.2764	1155.27	-5.542	11.4495	Aerial Stem Leaf
56	Procyanidin C1 3',3''-di-O-gallate	MolDiscovery	Phenylpropanoids and	Flavonoids	Biflavonoids and	C59H46O26	[M + H]+	1171.2349	1171.23	-4.2106	13.3533	Aerial Stem Leaf

			polyketides		polyflavonoids							
57	Lopac-P-101	CSI:FingerID	Nucleosides, nucleotides, and analogues	Purine nucleosides		C16H18N6O4	[M + H]+	359.1462	359.1486	6.8352	23.8542	Leaf
58	6-[(4,6-diamino-3-{[3-amino-6-(aminomethyl)-4,5-dihydroxyoxan-2-yl]oxy}-2-hydroxycyclohexyl)oxy]-2-(hydroxymethyl)-3-methyl-4-(methylamino)oxane-3,5-diol	CSI:FingerID	Organic oxygen compounds	Organooxygen compounds	Carbohydrates and carbohydrate conjugates	C20H41N5O10	[M + Na]+	534.2751	534.2732	-3.4898	21.4507	Leaf
59	Luminmycin B	CSI:FingerID	Organic acids and derivatives	Carboxylic acids and derivatives	Amino acids, peptides, and analogues	C27H48N4O7	[M - H2O + H]+	523.3496	523.346	-6.7819	18.2089	Stem Leaf
