# Supplementary material for: Metabolomic Characterization of Phoradendron brachystachyum Mistletoe and In-Silico and In-Vitro Investigation of Its Therapeutic Potential in Metabolic Disorders
Source: Plants (Basel). 2023 Jul 22;12(14):2729. doi: 10.3390/plants12142729 (PMC10383180; doi:10.3390/plants12142729)
Supplement: Supplementary file 1 [file plants-12-02729-s001.zip › 05 SUPPORTING_INFORMATION_TOJI_LAMI.pdf]

SUPPORTING INFORMATION

Supplemental experimental methods

LC-MS2 data processing

We followed a similar methodology previously described by our group [1] with some modifications. For task 1, raw LC-MS2 datasets (.d scan format) were converted to .mzXML using MSconvert (ProteoWizard) [2]. Mass detection, chromatogram building and deconvolution (ADAP algorithm), isotopic assignment, feature alignment, and gap-filling (to detect features missed during the initial alignment) were performed in MZmine Version 2.53 [3]. Features or peaks detected in blank samples (water: acetonitrile at 95:5 v:v, 0.1% formic) were considered contaminants and eliminated from samples by manual inspection. The final list containing the filtered features was exported as a .csv file (quantification table). The overlapping pattern of the features among the plant's anatomical sites was determined by UpSet plotting using the UpSetR app (open-source R package) [4,5]. For task two, .mzXML files were analyzed using the global natural products social molecular network (GNPS, https://gnps.ucsd.edu) to perform classical molecular networking and automated structural annotation (Metabolomics Standards Initiative [MSI] classification level 2) [6] (against GNPS public spectral libraries) [6].The parameters used within the GNPS were precursor ion and product ion mass tolerance of 0.02 Da. The molecular network was created using a minimum cosine score of 0.6 and a minimum of 4 peaks matched. The spectra in the network were searched against GNPS spectral libraries using a minimum cosine score of 0.6 and at least 4 matched peaks as filters. MS cluster option was selected. To expand the annotation of the metabolites not automatically retrieved by spectral matching, we employed MolDiscovery [7] and SIRIUS [8] *in-silico* annotation tools (MSI, level 3). For MolDiscovery, the clustered .mgf file retrieved from Classical Molecular Networking in the GNPS platform was analyzed

using the following parameters: precursor and fragment ion mass tolerance, 0.02 Da; max charge, 2, predefined DB, AllDB (720K compounds). For SIRIUS, the .mzXML files were imported into SIRIUS software GIU version 4.9.12 [8] to assign a chemical formula [9], molecular structure [10] and chemical class [11].We only selected compounds <860 Da. Annotations with a COSMIC Score> 0.65 were only considered. Common contaminants (e.g., plasticizers, silanones) and halogen- and boron-containing metabolites were removed from the analysis. The overlapping pattern of the chemical classes among the plant's anatomical sites was determined by UpSet plotting using the ComplexUpSet package based on the UpSetR package in R [4,5]. Molecular structures were drawn using ChemDraw Professional version 16.0.1.4.

1. Contreras-Angulo, L.A.; Moreno-Ulloa, A.; Carballo-Castañeda, R.A.; León-Felix, J.; Romero-Quintana, J.G.; Aguilar-Medina, M.; Ramos-Payán, R.; Heredia, J.B. Metabolomic Analysis of Phytochemical Compounds from Agricultural Residues of Eggplant (Solanum melongena L.). *Molecules* **2022**, *27*, 7013, doi:https://doi.org/10.3390/molecules27207013.
2. Holman, J.D.; Tabb, D.L.; Mallick, P. Employing ProteoWizard to convert raw mass spectrometry data. **2014**, *46*, 13.24. 11-13.24. 19, doi:https://10.1002/0471250953.bi1324s46
3. Pluskal, T.; Castillo, S.; Villar-Briones, A.; Orešič, M. MZmine 2: modular framework for processing, visualizing, and analyzing mass spectrometry-based molecular profile data. **2010**, *11*, 1-11, doi:https://10.1186/1471-2105-11-395
4. Lex, A.; Gehlenborg, N.; Strobelt, H.; Vuillemot, R.; Pfister, H. UpSet: visualization of intersecting sets. **2014**, *20*, 1983-1992, doi:https://10.1109/TVCG.2014.2346248
5. Conway, J.R.; Lex, A.; Gehlenborg, N. UpSetR: an R package for the visualization of intersecting sets and their properties. **2017**, doi:https://10.1093/bioinformatics/btx364
6. Aron, A.T.; Gentry, E.C.; McPhail, K.L.; Nothias, L.-F.; Nothias-Esposito, M.; Bouslimani, A.; Petras, D.; Gauglitz, J.M.; Sikora, N.; Vargas, F. Reproducible molecular networking of untargeted mass spectrometry data using GNPS. **2020**, *15*, 1954-1991, doi:https://10.1038/s41596-020-0317-5
7. Cao, L.; Guler, M.; Tagirdzhanov, A.; Lee, Y.-Y.; Gurevich, A.; Mohimani, H. MolDiscovery: Learning mass spectrometry fragmentation of small molecules. **2021**, *12*, 3718, doi:https://10.1038/s41467-021-23986-0

8. Dührkop, K.; Fleischauer, M.; Ludwig, M.; Aksenov, A.A.; Melnik, A.V.; Meusel, M.; Dorrestein, P.C.; Rousu, J.; Böcker, S. SIRIUS 4: a rapid tool for turning tandem mass spectra into metabolite structure information. **2019**, *16*, 299-302, doi:https://10.1038/s41592-019-0344-8
9. Ludwig, M.; Nothias, L.-F.; Dührkop, K.; Koester, I.; Fleischauer, M.; Hoffmann, M.A.; Petras, D.; Vargas, F.; Morsy, M.; Aluwihare, L. Database-independent molecular formula annotation using Gibbs sampling through ZODIAC. **2020**, *2*, 629-641, doi:https://doi.org/10.1038/s42256-020-00234-6.
10. Hoffmann, M.A.; Nothias, L.-F.; Ludwig, M.; Fleischauer, M.; Gentry, E.C.; Witting, M.; Dorrestein, P.C.; Dührkop, K.; Böcker, S. High-confidence structural annotation of metabolites absent from spectral libraries. **2022**, *40*, 411-421, doi:https://10.1038/s41587-021-01045-9
11. Dührkop, K.; Nothias, L.-F.; Fleischauer, M.; Reher, R.; Ludwig, M.; Hoffmann, M.A.; Petras, D.; Gerwick, W.H.; Rousu, J.; Dorrestein, P.C. Systematic classification of unknown metabolites using high-resolution fragmentation mass spectra. **2021**, *39*, 462-471, doi:https://10.1038/s41587-020-0740-8
